# Editorial: Risks of “cyber-relationships” in adolescents and young people

**DOI:** 10.3389/fpsyg.2023.1118736

**Published:** 2023-01-17

**Authors:** Joana Jaureguizar, Iratxe Redondo, Juan Manuel Machimbarrena, Sebastian Wachs

**Affiliations:** ^1^Department of Developmental and Educational Psychology, University of the Basque Country, Leioa, Spain; ^2^Department of Clinical and Health Psychology and Research Methodology, University of the Basque Country, Donostia-San Sebastián, Spain; ^3^Department of Educational Sciences, University of Potsdam, Potsdam, Germany

**Keywords:** social media, adolescent and youth, cyber violence, internet gaming disorder (IGD), cyberbullying, internet use and impact

How young people and adolescents interact today has changed considerably concerning the forms of interaction of a few decades ago, with the Internet and social media playing a leading role in their daily lives. Nearly 90 % of 13–17 year old adolescents use at least one social media platform to some degree (Anderson and Jiang, 2018). Thus, young people and adolescents use virtual social networks to form groups or initiate social relationships (Martínez-Ferrer and Moreno, 2017), to maintain contact with their peers (Senkbeil, 2018), and even to initiate and maintain their first romantic relationships (Lykens et al., 2019).

The immediacy, accessibility, lack of limits, and lack of real exposure can facilitate the emergence of new problems that, although they also existed before (e.g., bullying, addictions, intimate partner violence), now take new forms (e.g., cyberbullying, Internet addiction, generalized pathological internet use, Internet gaming disorder, cyber dating violence). The Diagnostic and Statistical Manual of Mental Disorders (DSM) has also been updated and has included in its manual, for example, internet gaming disorder as a disorder that needs further analysis in its latest edition (DSM-5; American Psychiatric Association., 2013). Thus, new risks for young people and adolescents potentially affecting their wellbeing are arising (George and Odgers, 2015). Some of these internet risks (e.g., cyberbullying, cyber dating abuse) could be understood as psychosocial problems that are initiated and maintained in an online context but keep a reciprocal and bidirectional relationship with the person's offline reality (Machimbarrena et al., 2018). These risks can have severe consequences for victims and aggressors, who often present internalizing and externalizing problems (Montiel et al., 2015; Garaigordobil and Machimbarrena, 2019; Azhari et al., 2022; Gámez-Guadix et al., 2022; Wachs et al., 2022), lower leves of health related quality of life (González-Cabrera et al., 2018), suicidal ideation (Quintana-Orts et al., 2022) and interference in academic, social, and family life (Cerniglia et al., 2016; Wright and Wachs, 2021).

This Research Topic aimed to study the risks of these new forms of interaction, the so-called “cyber-relationships”. Thus, this Research Topic brings together a series of scientific articles on these new realities. These articles are grouped into four main blocks: (1) the inappropriate use of the Internet and social networks; (2) Internet Gaming Disorder; (3) cyberbullying; and (4) instruments for evaluating these new forms of relationships.

As far as pathological Internet use is concerned, this Research Topic includes five articles. The article by Lin et al. about the influence of interpersonal sensitivity on smartphone addiction concluded that the fear of missing out and relational self-construal in college students played a moderated mediation effect on the relationship between smartphone addiction and the personality trait of interpersonal sensitivity (constantly worrying about negative social evaluation). This study has exciting implications for clinical practice and educational practitioners. Equally interesting in terms of clinical implications are the results of the brief research report of Moretta and Buodo about the relationship between affective and obsessive-compulsive symptoms in internet use disorder, where a strong and positive association between mild-moderate Internet use disorder and obsessive-compulsive symptoms was found. These authors found that hoarding (uncontrollable accumulation of digital information resulting in stress symptoms), obsessing, and depression symptoms were positively linked to Internet use disorder severity.

The study by Wang et al. explores problem network behavior and analyses the effect of factors such as adolescents' shyness, gender, and loneliness. It provides suggestions for the rational use of these networks. Loneliness was also a variable of interest in the study by Wang et al., who studied the mediating roles of internet gaming disorder, social network use, and the generalized pathological internet use (GPIU) in the relationship between depression and loneliness in Chinese adolescents, and found that loneliness could predict depression through the Internet gaming disorder and social network use to GPIU. Tian et al. conducted a study about the association between generalized and specific problematic Internet use to further deepen our understanding of pathological Internet use. They found that Internet gaming was the most critical predictor of GPIU: the effect of Internet gaming on GPIU was larger than the effects of online shopping, online pornography, and SNS usage.

Internet gaming is another main block of the present Topic, where two articles have been included. Firstly, Zhang et al. aim to analyze whether Internet Gaming Disorder (IGD) is associated with a high level of mind-wandering (defined as task-irrelevant thoughts) and how social anxiety plays a role in this relationship. The results suggested that excessive gaming behavior might increase mind-wandering and that social anxiety partially mediated this relationship. The second study is the article by Broman et al., developed in seven European countries about gambling, gaming, and problematic Internet behavior, to explore if these problems are affected by sexual orientation status. They found no differences among heterosexual and sexual minority men, but sexual minority women were associated with problematic gambling and gaming behavior.

The third block of the Research Topic is cyberbullying, which includes six articles. The article by Fernández-Antelo and Cuadrado-Gordillo brings a novel approach to this phenomenon since it studies the perception of aggressors and victims about cyberbullying and helps us understand better the factors and mechanisms that are involved in it. In turn, Zhong et al. explore the influencing factors of cyberbullying among Chinese college students, such as students' personal background, average daily Internet use, personality traits, emotions, and digital citizenship. They found that all these factors were relevant in cyberbullying perpetration and victimization, leading to interesting cyberbullying intervention and governance processes. The importance of adequate monitoring, adequate training of students, and adult supervision are emphasized. In this line, Ngo et al. explore the mediating effects of social support (family, peers, and teachers) on the associations between cyberbullying and psychological problems (anxiety, depression, and stress). They found a protective mediation effect of family support on the association between cyberbullying (experience and observation) and psychological problems.

Contrary to expectations, peer and teacher support did not protect students from experiencing or witnessing cyberbullying. Thus, positive child-parent relationships are critical in adolescence, so they feel they can confide in their parents when they suffer violence from their peers or even when they observe it. Many studies focus on bystanders as crucial figures in maintaining and curbing harassment. This is the case of the other three studies included in this Research Topic: the study by Barlinska et al., the review study by Polanco and Salvo-Garrido, and the article by Leung. On the one hand, Barlinska et al. concluded that activating more cognitive empathy among bystanders is crucial because it increases the likelihood of intervening in bystander behavior. In this line, Leung also emphasizes the role of empathy for bystanders, as he found that past cyberbullying victimization was positively related to cyber-defending behavior, which the activation of a greater empathy might explain. However, Polanco and Salvo-Garrido conclude that bystanders are not a homogeneous group in terms of characteristics and behavior and that contextual variables should also be taken into account since cyberbullying is a phenomenon that requires a multidisciplinary approach.

Lastly, the fourth block of articles of the Research Topic comprises two articles on evaluation instruments. On the one hand, Gaete et al. present a validation of the Revised Olweus Bully/Victim Questionnaire (OBVQ-R) among adolescents in Chile. This self-report questionnaire was initially used in different countries and had good psychometric properties. On the other hand, Persram et al. developed a new comprehensive teen dating violence victimization measure and evaluated its psychometric properties in Canadian adolescents.

In short, this Research Topic brings together a series of exciting articles that broaden our knowledge about the new forms of online relationships among young people and adolescents, with interesting practical implications, which open new avenues of research on online risk among young people and the development of intervention strategies to ensure young people's wellbeing in the 21st century.

## Author contributions

All authors have been the editors of the Research Topic, have contributed to the editorial article, and contributed to manuscript revision, read, and approved the submitted version.
